# Frequency and Distribution of Refractive Error in Adult Life: Methodology and Findings of the UK Biobank Study

**DOI:** 10.1371/journal.pone.0139780

**Published:** 2015-10-02

**Authors:** Phillippa M. Cumberland, Yanchun Bao, Pirro G. Hysi, Paul J. Foster, Christopher J. Hammond, Jugnoo S. Rahi, UK Biobank Eyes & Vision Consortium

**Affiliations:** 1 Life Course Epidemiology and Biostatistics Section, University College London (UCL) Institute of Child Health, London, United Kingdom; 2 Ulverscroft Vision Research Group, UCL Institute of Child Health, London, United Kingdom; 3 Department of Ophthalmology, King’s College London, St Thomas’ Hospital Campus, London, United Kingdom; 4 National Institute for Health Research (NIHR) Biomedical Research Centre at Moorfields Eye Hospital NHS Foundation Trust and UCL Institute of Ophthalmology, London, United Kingdom; 5 Department of Twin Research and Genetic Epidemiology, King’s College London, St Thomas’ Hospital Campus, London, United Kingdom; 6 Great Ormond Street Hospital for Children NHS Foundation Trust, London, United Kingdom; Medical College of Soochow University, CHINA

## Abstract

**Purpose:**

To report the methodology and findings of a large scale investigation of burden and distribution of refractive error, from a contemporary and ethnically diverse study of health and disease in adults, in the UK.

**Methods:**

U K Biobank, a unique contemporary resource for the study of health and disease, recruited more than half a million people aged 40–69 years. A subsample of 107,452 subjects undertook an enhanced ophthalmic examination which provided autorefraction data (a measure of refractive error). Refractive error status was categorised using the mean spherical equivalent refraction measure. Information on socio-demographic factors (age, gender, ethnicity, educational qualifications and accommodation tenure) was reported at the time of recruitment by questionnaire and face-to-face interview.

**Results:**

Fifty four percent of participants aged 40–69 years had refractive error. Specifically 27% had myopia (4% high myopia), which was more common amongst younger people, those of higher socio-economic status, higher educational attainment, or of White or Chinese ethnicity. The frequency of hypermetropia increased with age (7% at 40–44 years increasing to 46% at 65–69 years), was higher in women and its severity was associated with ethnicity (moderate or high hypermetropia at least 30% less likely in non-White ethnic groups compared to White).

**Conclusions:**

Refractive error is a significant public health issue for the UK and this study provides contemporary data on adults for planning services, health economic modelling and monitoring of secular trends. Further investigation of risk factors is necessary to inform strategies for prevention. There is scope to do this through the planned longitudinal extension of the UK Biobank study.

## Introduction

Refractive error is common and a focus of global initiatives against avoidable (preventable or treatable) causes of impaired vision[1–4] particularly in low-income countries where it is more frequently undetected or untreated and thus associated with loss of productivity[5]. The financial costs of ‘treatment’ (corrective glasses or contact lenses or more recently refractive surgery) confer a substantial economic burden on health services and individuals [6,7]. Furthermore, those with high myopia (defined as spherical equivalent -6 diopters (D) or worse) are at increased risk of developing sight-threatening complications e.g. glaucoma, cataract or retinal detachment [8–10]. The prevalence of myopia (short-sightedness) is documented to have increased significantly over the last three decades [11–13] in some countries, notably in East Asia, with a significant shift in the distribution towards both increasing severity and younger age at onset[14,15]. In addition, with an ageing global population, the burden of presbyopia (age-related hypermetropic shift in refraction) is set to increase. Knowledge of the frequency and distribution of refractive error in contemporary and ethnically diverse populations in industrialised countries is incomplete but necessary for planning of services.

UK Biobank (UKBB), the world’s largest single resource for the study of health and disease, recruited more than half a million adults between 2006–2010[16,17]. In 2009, the protocol was extended to include an enhanced ophthalmic examination which provided autorefraction data (i.e. a measure of refractive error) on a subsample of subjects. This sub-study presents a unique resource for the study of refractive error in an ethnically diverse, contemporary adult population in an industrialised country setting. We report here the methodology of the study and the baseline frequencies of myopia and hypermetropia and associations with key socio-demographic factors.

## Methods

### Study population

UK Biobank recruited 502,682 subjects, aged between 40 and 69 years, from all those registered with the UK National Health Service and living within a 25 miles radius of one of the 22 study assessment centres. Participants reported on their life-style, environment and medical history via touchscreen and face-to-face interviews and a wide range of physical measures were taken[17]. At 6 recruitment centres, 5 in England and 1 in Wales, 117,279 (23.3%) participants attended for an enhanced ophthalmic assessment, which included autorefraction. Participants who reported having any eye surgery in the preceding 4 weeks or a current eye infection were not eligible for assessment (S1 Fig).

### Outcome measures and definitions of refractive error

Non-cycloplegic autorefraction was carried out using the Tomey RC 5000 auto refkeratometer (Tomey Corp., Nagoya, Japan). The right eye was tested first, up to 10 refractive error measurements for each eye were taken and the most representative result automatically recorded. The autorefractor recorded a reliability score, 0 to 9 (smaller scores indicating more reliable measurements), for each measurement, with a score of ≤4 defining reliability.

To avoid misclassification and erroneous estimation of frequency, some individuals were excluded from the analysis. Firstly, those whose autorefraction results in combination with self-report of wearing glasses or contact lenses (optical correction) and eye conditions indicated that their refractive error was likely to be secondary to cataract rather than an independent primary disorder. Thus, individuals were excluded if they had low/mild myopia with self-reported diagnosis of cataract as well as either report of not wearing glasses or contact lenses or report of first wearing optical correction after age 30 years or if there was no information on age of first wearing optical correction. Secondly, individuals were excluded if they reported prior bilateral cataract surgery, refractive laser surgery, vitrectomy or retinal detachment i.e. conditions/treatment which meant that their primary refractive error status could not be determined.

Spherical equivalent (SE) (algebraic sum in diopters (D), sphere+0.5cylinder) was used to categorise refractive error using conventional thresholds; emmetropia (SE -0.99D to +0.99D), low primary myopia (SE -1.0D to -2.99D), moderate primary myopia (SE -3.0D to -5.99D), high primary myopia (SE -6.0D or more extreme), low hypermetropia (SE +1.0D to +2.99D) and moderate/high hypermetropia (SE + 3.0D or more extreme).

### Available UK Biobank data

At the time of recruitment all participants completed a touchscreen questionnaire, reporting on demographic, socio-economic and medical factors (see listing: Table 1). They then had a face-to-face interview which was informed by responses to the touchscreen questionnaire and an ‘aide-memoire’ questionnaire, completed before the visit, to assist in answering questions on medications, medical history, family history of diseases and early life events. Specifically, the face-to-face interview enabled report of ‘other’ eye conditions/cancers/surgery not included in the response list of the lead touchscreen questions on medical history. Information about history of any eye condition and treatment received was collated on each subject using all available data.

**Table 1:** Demographic, socio-economic and other factors.

Factors	Description and/or categorisation
At recruitment: age and gender	
Touchscreen questionnaire and face-to-face interview	
**Ethnicity**	White, mixed (white with Black/Black British or Asian/Asian British or other ethnic group mix), Asian or Asian British (Indian, Pakistani or Bangladeshi), Black or Black British (Caribbean or African), Chinese or Other
**Highest educational attainment**	No qualifications, State school examinations at 16 years of age (‘O’ levels), at 18 years (‘A’ levels) or University/other professional qualification
**Accommodation tenure**	Council rental, private rental, home-ownership with a mortgage or outright ownership
**Wearing glasses/contact lenses**	Do you wear glasses or contact lenses to correct your vision? (yes, no, prefer not to answer). What age did you first start wearing glasses or contact lenses? "Why were you prescribed glasses/contacts? (You can select more than one answer)". For short-sightedness, i.e. only or mainly for distance viewing such as driving, cinema etc. (called 'myopia'). For long-sightedness, i.e. for distance and near, but particularly for near tasks like reading (called 'hypermetropia'). For just reading/near work as you are getting older (called 'presbyopia'). For 'astigmatism'. For a 'squint' or 'turn' in an eye since childhood (called 'strabismus'). For a 'lazy' eye or an eye with poor vision since childhood (called 'amblyopia'). Other eye condition. Do not know. Prefer not to answer.
**Common eye conditions and age of diagnosis**	Has a doctor told you that you have any of the following problems with your eyes? e.g. diabetes-related eye disease, glaucoma, trauma, cataract, macular degeneration or other serious eye condition
**Ever had eye surgery**	Eye condition and age of surgery
**Non-cancerous or cancerous illness**	Eye and eyelid problems e.g. retinal detachment **or** eye and/or adnexal cancer including retinoblastoma
**Handedness (laterality)**	Right-handed, left-handed or use both right and left hands equally

### Statistical Methods

#### Frequency of refractive errors

Mean spherical equivalent of the two eyes for each individual was used, except for participants who had only one eye measurement. In addition for this analysis, to avoid misclassification of individuals by category of refractive error, participants who had the following forms of inter-ocular discordance in refraction were excluded: i) one eye hypermetropic and the other eye myopic, or ii) one eye high/severe refractive error and the other eye emmetropic or iii) one eye mildly myopic/hypermetropic and the other eye highly myopic/hypermetropic with a difference of at least 10 D in SE between the two eyes.

#### Associations between refractive error and socio-demographic factors

Bivariate logistic regression was used to model the paired data (2 eyes), with emmetropia (SE -0.99D to +0.99D) as the reference category. An indicator for laterality was used in the model to account for any difference between the paired data and the interactions of eye indicator with the socio-demographic factors were investigated.[18]. Models were adjusted for test centre to account for any heterogeneity among centres and robust variance estimates were used to adjust for correlation within test centre. Multivariable analyses, using backward stepwise regression, included factors that were significant at a 10% level in initial univariable analysis. Factors were retained in the multivariable model if they altered the risk ratio estimate by more than 10% or if they were independently associated at a 5% significance level.

Paired t-tests were used to test for the difference between SE in right and left eyes.

All analyses were carried out using Stata 13.0 (StataCorp, College Station, Texas).

UK Biobank has approval from the North West Multi-Centre Research Ethics committee, which covers the UK. It also sought the approval in England and Wales from the Patient Information Advisory Group for gaining access to information that would allow it to invite people to participate. PIAG has since been replaced by the National Information Governance Board for Health & Social Care. In Scotland, UK Biobank has approval from the Community Health Index Advisory Group. Recruitment into the UK Biobank study was by written consent.

## Results

### Participation and final study sample

Of 115,795 individuals eligible for an ophthalmic examination, 1,864 subjects were not assessed for various reasons e.g. self-report of visual impairment or unwillingness to remove contact lenses and in 731 no reading was obtained due to equipment failure (S1 Fig). As described above, those who self-reported having eye conditions known to affect refractive error status (n = 4,887) and those who had low myopia considered to be secondary to cataract (n = 129), were excluded as were 732 subjects who had highly discordant refraction measures. Thus data on 107,452 subjects were analysed; 105,969 (98.6%) with both eyes measured, 725 (0.7%) with only a right and 758 (0.7%) with only a left eye measured.

### Representativeness

Based on a comparison with the UK Census 2011 data [https://www.nomisweb.co.uk/census/2011], the study population is older and has fewer males, but the ethnic distribution is comparable (90% White, 3.8% Asian/Asian British including 0.5% Chinese, 3.5% Black/ Black British, 0.9% of Mixed and 1.5% Other ethnicity). However, on average, the study population is more affluent with a higher proportion owning their homes and having higher educational qualifications. Those excluded from the analysis were more likely to be 60–69 years compared to 40–49 years (odds ratio 1.6 [95% confidence interval 1.5, 1.7] and, independently, of Asian/ Asian British, Black/Black British or Other ethnicity (OR 1.5 [1.4, 1.7], OR 1.3 [1.2, 1.4] or OR 1.5 [1.2, 1.7] respectively), compared to those of White ethnicity (S1 Table).

### Frequency of refractive errors

In this population, the mean value of spherical equivalent was -0.29D [-0.31, -0.27] with range -23.5D to +13.9D. The frequency of myopia was 26.9% [26.6, 27.1], of hypermetropia 27.6% [27.3, 27.8] and 48,964 (45.6% [45.3, 45.9]) subjects were classified as emmetropic. Categorising refractive error by severity, 4.0% [3.9, 4.1] of the study population were high myopes, 9.5% [9.4, 9.7] were moderately myopic and 13.3% [13.1, 13.5] had low myopia; 5.9% [5.8, 6.0] were high/moderate hypermetropes, and 21.7% [21.4, 21.9] were mildly hypermetropic. The frequency of myopia, defined as ≤-0.5D, was 33.5% [33.3, 33.9] and hypermetropia, defined as ≥+0.5D, 39.7% [39.4, 40.0].

### Distribution of refractive error frequency and associations with socio-demographic factors

**Fig 1:**
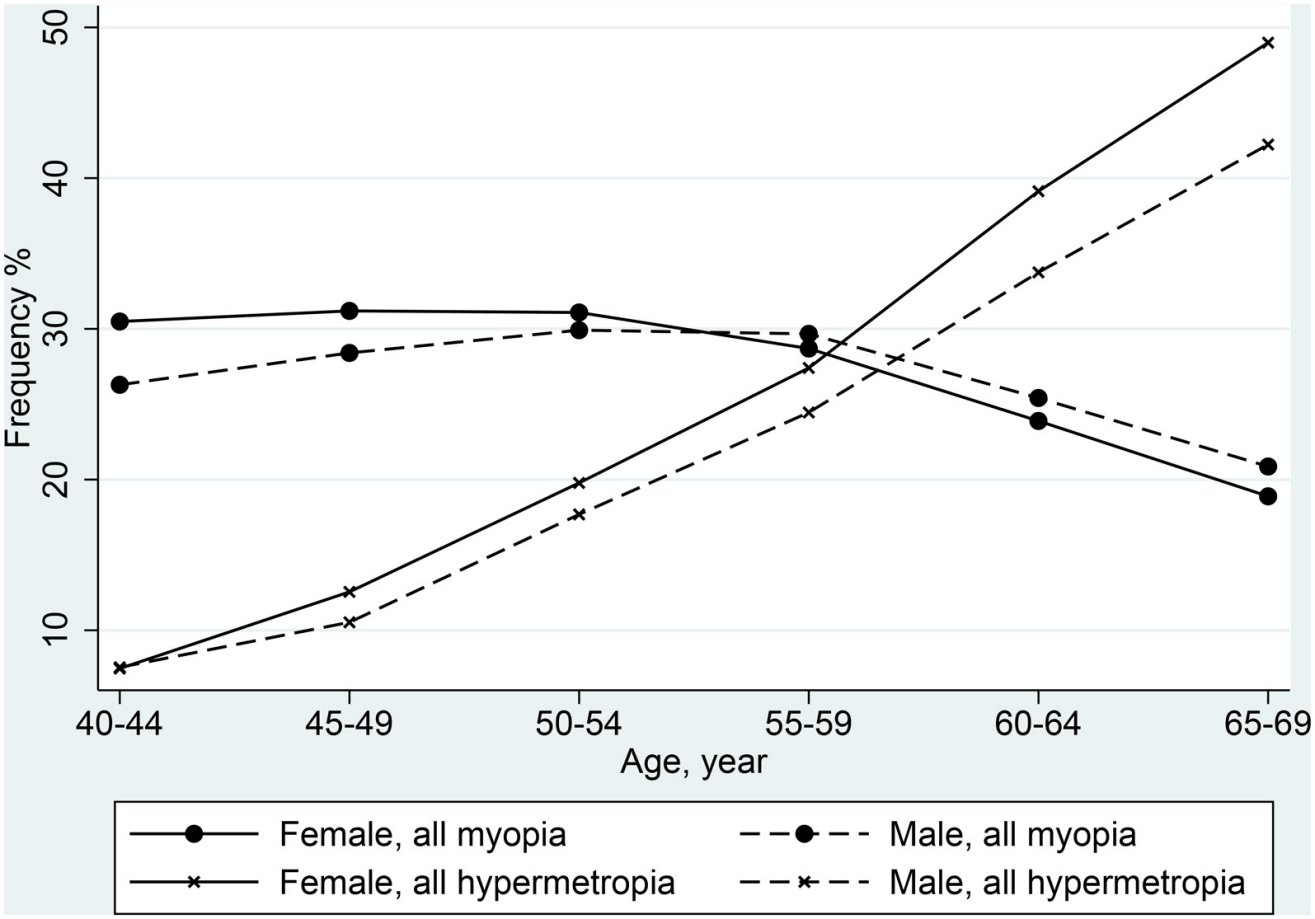
Frequency of refractive errors by 5-year age band and gender.

**Fig 2:**
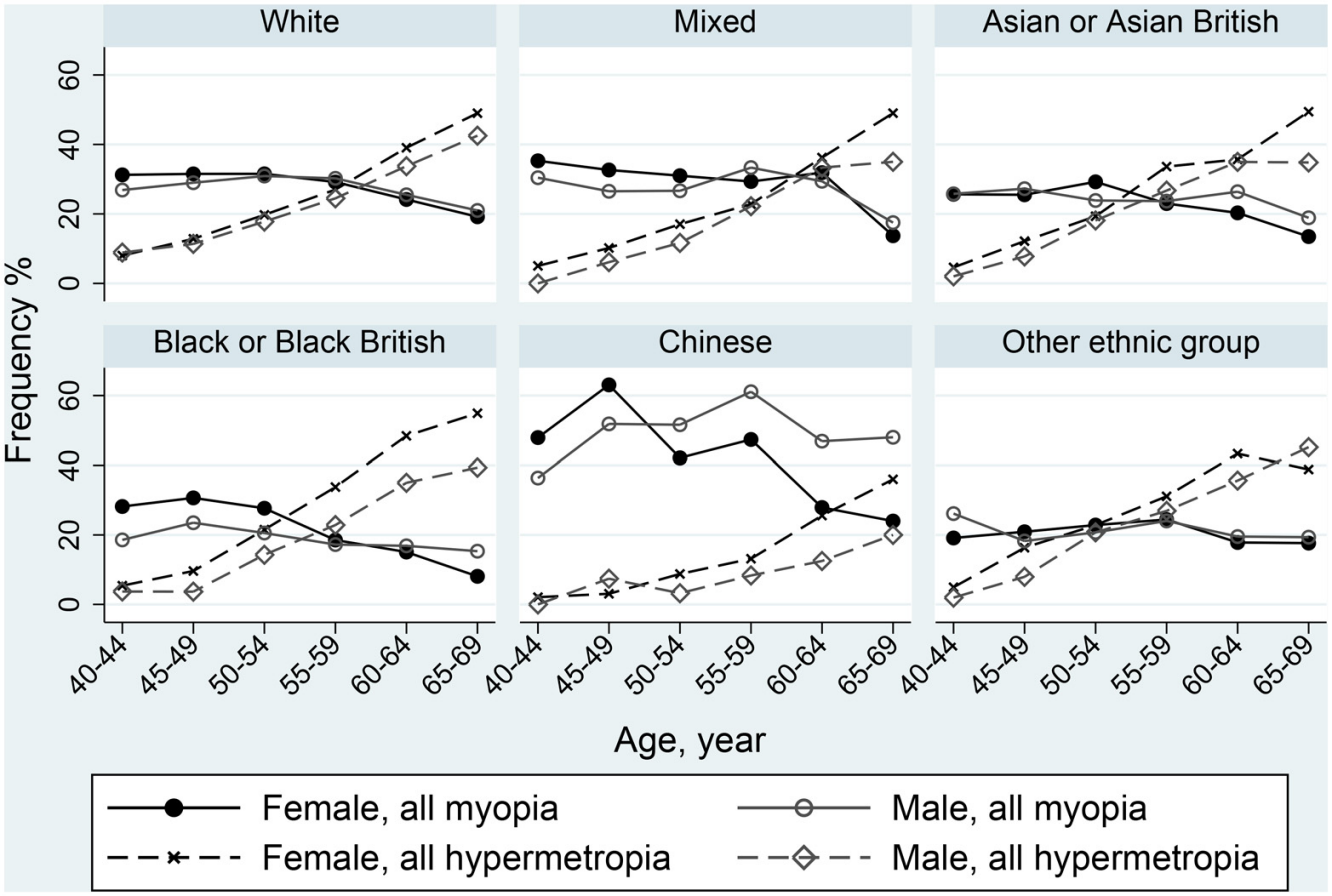
Frequency of all myopia and all hypermetropia by age, gender and ethnicity.

The distributions of myopia frequency by age, gender and other demographic factors are given in Table 2 and Fig 1. The Chinese ethnic group had the highest overall frequency of myopia (47.4% [42.9% 51.8%]), (50.9% [43.4% 58.3%] in men and 45.4% [39.9% 51.0%] in women), nearly two-fold higher overall than other ethnic groups and over 3-fold higher for high myopia (Fig 2). Men of Black/Black British ethnicity were least likely to have myopia, 20.8% [18.8, 22.9] compared to 25.0% [23.3, 26.9] in Black/Black British women and 27.1% [26.8, 27.3] in those of White ethnicity. In fully adjusted multivariable analyses comparing each category of myopia to those with emmetropia, women were more likely than men to have moderate or high myopia, (OR 1.15 [1.10 1.20] and OR 1.24 [1.16 1.31]) (Table 3). Increasing educational achievement and accommodation tenure status were significantly associated with higher frequency of myopia, the strength of the association increasing with severity of myopia.

**Table 2:** Distribution of refractive errors by key socio-demographic factors.

N = 107,452	Total		Myopia (≤-1 diopter)			Hypermetropia (≥+1 diopter)		
	n	%	n	% [95% confidence interval]		n	% [95% confidence interval]	
All	107,452		28,857	26.9	[26.6, 27.1]	29,631	27.6	[27.3, 27.8]
Gender								
Male	49,000	45.6	13,116	26.8	[26.4, 27.2]	12,745	26.0	[25.6, 26.4]
Female	58,452	54.4	15,741	26.9	[26.6, 27.3]	16,886	28.9	[28.5, 29.3]
Age group (years)								
40–44	10,857	10.1	3,169	29.2	[28.3, 30.1]	807	7.4	[7.0, 7.9]
45–49	13,925	13.0	4,258	30.6	[29.8, 31.3]	1,610	11.6	[11.0, 12.1]
50–54	16,116	15.0	4,996	31.0	[30.3, 31.7]	3,026	18.8	[18.2, 19.4]
55–59	18,737	17.4	5,525	29.5	[28.8, 30.1]	4,867	26.0	[25.4, 26.6]
60–64	27,053	25.2	6,729	24.9	[24.4, 25.4]	9,881	36.6	[36.0, 37.1]
65–69	20,764	19.3	4,180	20.1	[19.6, 20.7]	9,440	45.5	[44.8, 46.2]
Highest educational qualification*								
None	15,776	14.9	2,063	13.1	[12.6, 13.6]	6,762	42.9	[42.1, 43.7]
‘O’ level	28,275	26.7	6,845	24.2	[23.7, 24.7]	7,579	26.8	[26.3, 27.3]
‘A’ level	19,162	18.1	5,103	26.6	[26.0, 27.3]	5,085	26.6	[25.9, 27.2]
Higher-level	42,773	40.4	14,624	34.2	[33.7, 34.6]	9,701	22.7	[22.3, 23.1]
Accommodation tenure								
Council rental	7,301	6.9	1,425	19.5	[18.6, 20.4]	2,155	29.5	[28.5, 30.6]
Private rental	4,351	4.1	1,026	23.6	[22.3, 24.9]	997	22.9	[21.7, 24.2]
Own with mortgage	37,858	35.9	10,984	29.0	[28.6, 29.5]	7,431	19.6	[19.2, 20.0]
Own	55,840	53.0	14,936	26.9	[26.3, 27.1]	18,445	33.1	[32.7, 33.4]
Ethnicity								
White	95,791	89.8	25,919	27.1	[26.8, 27.3]	27,243	28.5	[28.1, 28.7]
Mixed	974	0.9	290	29.8	[27.0, 32.7]	168	17.3	[15.0, 19.8]
Asian/British Asian	4,032	3.8	1,000	24.8	[23.5, 26.2]	847	21.0	[19.8, 22.3]
Black/British Black	3,759	3.5	876	23.3	[22.0, 24.7]	740	19.7	[18.5, 21.0]
Chinese	490	0.5	232	47.4	[42.9, 51.8]	53	10.8	[8.3, 13.9]
Other	1,627	1.5	355	21.8	[19.9, 23.9]	364	22.4	[20.4, 24.5]

* No qualifications, State school examinations at 16 years of age (‘O’ levels), at 18 years (‘A’ levels) or University/other professional qualification

Missing data: educational qualification: 1,466 (1.4%), accommodation tenure: 2,102 (2.0%), ethnicity: 779 (0.7%)

**Table 3:** Association of all myopia and myopia (low, moderate or high), by key socio-demographic factors.

	All myopia		High myopia		Moderate myopia		Low myopia		Emmetropia
	(SE ≤-1D)		(SE ≤-6D)		(SE -5.99 to -3D)		(SE -2.99 to -1D)		(SE -0.99 to 0.99D)
	N^+^	Odds Ratio	N^+^	Odds Ratio	N^+^	Odds Ratio	N^+^	Odds Ratio	N^+^
Factors	56,470	[95% CI]	8,528	[95% CI]	20,087	[95% CI]	27,855	[95% CI]	94,359
Eye^++^									
Right eye	28,462	**1**	4,290	1	10,114	1	14,058	1	47,403
Left eye	28,008	0.99 [0.98, 1.00]	4,238	0.99 [0.97, 1.02]	9,973	0.99 [0.98, 1.01]	13,797	0.99 [0.97, 1.01]	46,956
Age group (years)									
40–44	6160	1	915	1	2,214	1	3,031	1	12,975
45–49	8293	**1.13 [1.07, 1.20]**	1,301	**1.20 [1.06, 1.35]**	2,903	**1.10 [1.02, 1.19]**	4,089	**1.13 [1.06, 1.21]**	15,439
50–54	9819	**1.30 [1.23, 1.38]**	1,556	**1.35 [1.21, 1.52]**	3,465	**1.27 [1.17, 1.37]**	4,798	**1.30 [1.22, 1.39]**	15,622
55–59	10,831	**1.35 [1.28, 1.43]**	1,729	**1.37 [1.22, 1.54]**	3,823	**1.31 [1.21, 1.42]**	5,279	**1.37 [1.28, 1.46]**	16,212
60–64	13,123	**1.34 [1.27, 1.41]**	1,945	**1.25 [1.11, 1.41]**	4,667	**1.30 [1.20, 1.41]**	6,511	**1.37 [1.28, 1.46]**	20,352
65–69	8244	**1.31 [1.24, 1.40]**	1,082	1.10 [0.96, 1.25]	3,015	**1.33 [1.22, 1.45]**	4,147	**1.34 [1.25, 1.44]**	13,759
Gender^++^									
Male	25,661	1	3,539	1	8,779	1	13,343	1	44,459
Female	30,809	**1.07 [1.04, 1.11]**	4,989	**1.24 [1.16, 1.31]**	11,308	**1.15 [1.10, 1.20]**	14,512	0.97 [0.94, 1.00]	49,900
Highest educational qualification * ^++^									
No qualification	4081	1	437	1	1,251	1	2,393	1	13,381
‘O’ level	13,538	**1.70 [1.61, 1.80]**	1,786	**2.01 [1.75, 2.32]**	4,494	**1.84 [1.68, 2.01]**	7,258	**1.57 [1.47, 1.68]**	27,124
‘A’ level	10,062	**1.94 [1.83, 2.05]**	1,417	**2.43 [2.11, 2.82]**	3,534	**2.22 [2.03, 2.43]**	5,111	**1.68 [1.57, 1.80]**	7,511
Higher-level	28,789	**2.62 [2.49, 2.76]**	4,888	**3.90 [3.42, 4.46]**	10,808	**3.19 [2.94, 3.47]**	13,093	**2.07 [1.94, 2.20]**	36,343
Accommodation tenure^++^									
Council rental	2,761	1	351	1	879	1	1,531	1	7,157
Private rental	2,029	0.99 [0.90, 1.08]	291	1.02 [0.82, 1.26]	701	1.04 [0.91, 1.20]	1,035	0.96 [0.86, 1.09]	4,507
Own with mortgage	21,995	**1.22 [1.15, 1.30]**	3,225	**1.32 [1.13, 1.53]**	7,740	**1.31 [1.18, 1.44]**	11,029	**1.16 [1.08, 1.26]**	38,212
Own	29,687	**1.33 [1.25, 1.42]**	4,661	**1.65 [1.41, 1.93]**	10,766	**1.47 [1.33, 1.63]**	14,260	**1.19 [1.10, 1.29]**	44,483
Ethnicity^++^									
White	51,233	1	7,652	1	18,239	1	25,342	1	83,470
Mixed	576	1.00 [0.87, 1.15]	111	1.22 [0.94, 1.59]	206	1.00 [0.82, 1.22]	259	0.93 [0.78, 1.11]	950
Asian/Asian British	1,880	**0.78 [0.72, 0.84]**	309	**0.82 [0.70, 0.96]**	690	**0.81 [0.72, 0.90]**	881	**0.74 [0.68, 0.82]**	3,940
Black/Black British	1,675	**0.73 [0.67, 0.79]**	232	**0.65 [0.54, 0.78]**	536	**0.65 [0.57, 0.74]**	907	**0.79 [0.72, 0.88]**	3,965
Chinese	448	**1.86 [1.54, 2.23]**	132	**3.44 [2.62, 4.50]**	185	**2.11 [1.66, 2.67]**	131	1.15 [0.90, 1.47]	365
Other	658	**0.64 [0.57, 0.72]**	92	**0.57 [0.43, 0.76]**	231	**0.63 [0.52, 0.76]**	335	**0.68 [0.59, 0.79]**	1,669

* No qualifications, State school examinations at 16 years of age (‘O’ levels), at 18 years (‘A’ levels) or University/other professional qualification

^+^: Number of eyes;

^++^ model adjusted for eye laterality, gender, age (continuous), educational qualification, accommodation tenure, ethnicity and test centre.

There was a linear increase in the frequency of hypermetropia with age, with a steeper gradient in women than in men in all ethnic groups. (Table 2 & Fig 1). The associations between hypermetropia and both increasing age and being female were stronger in those with mild compared to more severe hypermetropia (Table 4). In mild hypermetropes there was an interaction between right/left eye and age, with a 15–25% reduced risk of mild hypermetropia in the left eye, with increasing age, compared to the right eye. Hypermetropes were less likely to have educational qualifications or own their own property, compared to emmetropes, these effects being stronger in those with more severe hypermetropia. Also, compared to those of White ethnicity, those with high/moderate hypermetropia were at least 30% less likely to be in a minority ethnic group.

**Table 4:** Association of all hypermetropia and hypermetropia (low or moderate/high), by key socio-demographic factors.

	All hypermetropia		High/moderate hypermetropia		Low hypermetropia		Emmetropia
	(SE ≥1D)		(SE ≥+3D)		(SE 1D to 2.99D)		(SE -0.99 to 0.99D)
	N^+^	Odds Ratio	N^+^	Odds Ratio	N^+^	Odds Ratio	N^+^
Factors	56,899	[95% CI]	12,794	[95% CI]	44,105	[95% CI]	94,359
Eye^++^							
Right eye	27,983	**1**	6,116	1	21,867	1	47,403
Left eye	28,916	**1.05 [1.04, 1.07]**	6,678	**1.11 [1.08, 1.13]**	22,238	**1.03 [1.02, 1.05]**	46,956
Age group (years)							
40–44	1,585	1	481	1	1,104	1	12,975
45–49	3,067	**1.61 [1.48, 1.75]**	870	**1.46 [1.26, 1.69]**	2,197	**1.67 [1.52, 1.83]**	15,439
50–54	5,833	**3.00 [2.78, 3.24]**	1,335	**2.14 [1.86, 2.46]**	4,498	**3.37 [3.09, 3.67]**	15,622
55–59	9,347	**4.62 [4.28, 4.98]**	1,897	**2.83 [2.47, 3.24]**	7,450	**5.40 [4.97, 5.87]**	16,212
60–64	19,024	**7.40 [6.87, 7.97]**	4,022	**4.55 [3.99, 5.19]**	15,002	**8.67 [7.98, 9.42]**	20,352
65–69	18,043	**10.31 [9.55, 11.13]**	4,189	**6.81 [5.95, 7.80]**	13,854	**11.85 [10.88, 12.90]**	13,759
Gender^++^							
Male	24,446	1	5,610	1	18,836	1	44,459
Female	32,453	**1.27 [1.23, 1.30]**	7,184	**1.21 [1.15, 1.27]**	25,269	**1.29 [1.25, 1.33]**	49,900
Highest educational qualification * ^++^							
No qualification	13,068	1	3,342	1	9,726	1	13,381
‘O’ level	14,790	**0.857 [0.83, 0.91]**	3,362	**0.76 [0.71, 0.82]**	11,428	**0.90 [0.86, 0.95]**	27,124
‘A’ level	10,005	**0.87 [0.83, 0.91]**	2,249	**0.76 [0.70, 0.82]**	7,756	**0.91 [0.87, 0.96]**	7,511
Higher-level	19,036	**0.80 [0.77, 0.83]**	3,841	**0.60 [0.60, 0.69]**	15,195	**0.85 [0.82, 0.89]**	36,343
Accommodation tenure^++^							
Council rental	4,116	1	1,035	1	3,081	1	7,157
Private rental	1,927	0.98 [0.89, 1.07]	396	**0.83 [0.70, 0.98]**	1,531	1.03 [0.93, 1.13]	4,507
Own with mortgage	14,660	**0.88 [0.83, 0.93]**	3,296	**0.76 [0.69, 0.85]**	11,364	**0.92 [0.86, 0.98]**	38,212
Own	36,196	**0.84 [0.79, 0.89]**	8,067	**0.76 [0.68, 0.83]**	28,129	**0.87 [0.82, 0.93]**	44,483
Ethnicity^++^							
White	52,949	1	12,335	1	40,614	1	83,470
Mixed	316	**0.77 (0.66 0.94)**	44	**0.47 (0.32 0.70)**	272	0.89 (0.74 1.07)	950
Asian	1,503	**0.85 (0.78 0.92)**	180	**0.44 (0.36 0.54)**	1,323	0.98 (0.89 1.06)	3,940
Black	1,373	**0.82 (0.75 0.89)**	135	**0.35 (0.28 0.43)**	1,238	0.78 (0.89 1.07)	3,965
Chinese	102	**0.53 (0.40 0.70)**	9	**0.21 (0.10 0.46)**	93	**0.63 (0.47 0.84)**	365
Other	656	0.89 (0.78 1.00)	91	**0.55 (0.41 0.72)**	565	1.00 (0.87 1.13)	1,669

* No qualifications, State school examinations at 16 years of age (‘O’ levels), at 18 years (‘A’ levels) or University/other professional qualification

^+^: Number of eyes;

^++^ model adjusted for eye laterality, gender, age (continuous), educational qualification, accommodation tenure, ethnicity and test centre.

### Difference in Spherical Equivalent between eyes and self-report of hand dominance

The mean spherical equivalent measurements were -0.31 D ± 2.71, standard deviation (SD), for the right eye and -0.27 D ± 2.73 SD for the left eye. Right eye measures were consistently more myopic with the mean difference between eyes varying from 0.07 D to 0.026 D across increasing age bands (differences at all ages significant; p ≤0.001) (Table 5).

**Table 5:** Comparison of mean spherical equivalent (SE) between paired left and right eyes, by age band.

	Age in 5-year age bands						
	40–44	45–49	50–54	55–59	60–64	60–69	Total
N (individuals)	10,739	13,802	15,922	18,500	26,640	20,366	105,969
Right eye SE	-0.901	-0.862	-0.704	-0.490	-0.009	0.431	-0.314
Left eye SE	-0.827	-0.792	-0.647	-0.439	0.026	0.457	-0.267
Left SE-Right SE	0.074	0.070	0.057	0.051	0.034	0.026	0.048
P-value (paired t-test)	<0.001	<0.001	<0.001	<0.001	<0.001	<0.001	<0.001

We investigated any association between spherical equivalent measurements and self-report of handedness as a proxy for ocular dominance. There was a significant difference between right eye SE -0.32D ± 2.71 and left eye SE -0.26D ± 2.72 (pair t test p<0.001) for right handed people but no such significance for left handed people, SE -0.31D ± 2.70 and SE -0.32D ± 2.76 (p = 0.356) for right and left eyes respectively. On average those who self-reported as ambidextrous had refractive error measures nearer zero with a significant difference between eyes, mean SE -0.24D ± 2.73 and -0.18D ± 2.71 (p = 0.004) for right and left eyes respectively.

## Discussion

We report the success of the UK Biobank (UKBB) study in delivering from a generic epidemiological study a high quality cross-sectional dataset on refractive error which is without parallel in scale. Our findings confirm that within the ethnically diverse and ageing population of the UK, refractive error is already a major public health issue and indicate that the burden can be anticipated to increase over time, given the associations with key socio-demographic factors.

Although sufficiently representative of the UK population, participants volunteered for the UKBB study, thus it does not comprise a true population sample, precluding formal population prevalence estimation. The enhanced ophthalmic examination was added to the protocol in 2009 after completion of data collection in Scotland, preventing investigation of regional differences, as the majority of subjects were from England. However, despite the contradictory effects of potential biases related to the study design and exclusion of individuals from the analysis, the overall frequency of primary myopia in the UKBB is consistent with other studies, taking into account differences in age range and threshold values for refractive errors between studies. Specifically, the frequency amongst White participants in is consistent with prior prevalence estimates in two recent comparable European studies[19,20]. The size and diversity of the UK Biobank sample, with almost complete data available for key variables, allow investigation of associations between all categories of refractive error and key socio-demographic factors and 6 main ethnic groups with effect estimates being generalizable to the wider population. The use of non-cyclopegic autorefraction in adults aged 40 years and older in the context of a population survey is considered robust[21,22]. Furthermore use of <-1D as the threshold for myopia reduced the potential misclassification of refractive errors due to non-cyclopegic assessment[23]. Detailed information on diagnosed eye/medical conditions recorded by participants, on a pre-visit proforma, was used to prompt individual questionnaire and interview responses. This will have reduced the potential for inaccuracy of recall and under-reporting. However, the lack of an expert clinical assessment of any medical condition precluded by the broad scope of the study meant that self-report could not be validated directly. The extensive data collected on UK Biobank participants enabled identification of those with secondary myopia and exclusion of these subjects from the analysis. Use of age at first optical correction as part of the definition of primary myopia could be problematic in settings where uncorrected refractive error is common but data on older adults in the UK suggest this is not a significant factor[24]. Thus frequency of and associations with *primary* myopia are reported here. We suggest accurate assignment of phenotype in this way is a particular strength of this study, avoiding misclassification in both epidemiological and genetic investigations.

There is limited information on the ethnic variation in prevalence of refractive errors in adults in European populations. Our findings in UKBB are broadly consistent with those reported in multi-ethnic study of a slightly older population in the United States [25] with respect to rates of myopia in Black (21.5% vs 22.3%) and White participants (31% vs 26.7%). The frequency of myopia in Asian/Asian British participants in UKBB, most of whom were Indian, is comparable with the frequency reported in Indians in Singapore[26]. Most notably the highest frequencies of myopia were in Chinese participants in UK Biobank and exceeded those reported in East Asian populations as well as in Chinese subjects in the US[25,27,28]. These findings, signalling that myopia is now well-established in the ethnically diverse population of the UK, provide baseline data for monitoring temporal trends.

In UKBB, myopia was more likely in women than men in the 40–59 age band but less likely in women in the older age group (60–69 years), echoing findings in the US and Australia[29,30]. We also found the positive associations between myopia and both higher socio-economic status and higher educational achievement that have reported previously [31–34]. However the cross-sectional nature of the present study makes it difficult to assess causality in relation to these socio-demographic factors or others of current interest such as near work activities, time spent in distance viewing and/or time spent outdoors [35–37].

The age related increase in hypermetropia and higher frequency in women found in UKBB confirms prior reports elsewhere[30,31,38,39]. The lower overall frequency of hypermetropia compared to other studies may reflect the upper age limit of 69 years, although the frequency of moderate/high hypermetropia was more comparable[19,25]. Notably, there are differences between low and moderate/high hypermetropia with respect to patterns of association with age, gender, ethnicity, and socio-economic factors. This may be attributable to fundamental differences between primary hypermetropia accounting for the moderate/high hypermetropia in the present study and the hyperopic shift that starts in mid-adult life and results in presbyopia i.e. low hypermetropia captured through non-cyclopegic refraction in the present study.

The finding in the present study that right eyes were on average more myopic has been reported, although not always convincingly, in other studies[19,31,33,38] and right eyes have also been reported to have greater axial length[40]. As right eyes were tested first in all individuals in UKBB, there is potential for a systematic measurement error. Equally, since the interocular difference decreased with increasing age, it may simply reflect the greater frequency of myopia in the younger and of hypermetropia in older age groups. There is some evidence of a correlation between eye dominance and myopia which is purported to account for the higher proportion of myopia in right eyes[41], and this in turn may underlie the higher frequency of retinal detachment in right eyes[40,42]. Although the correlation between eye dominance and handedness is not clear[43], in this large sample there are statistically significant differences in spherical equivalent by handedness. Absolute differences found are relatively small however we did find an interesting trend of right eyes being more myopic in right handed individuals, a weaker effect in those with no preference and no difference in left handed individuals. These findings serve as a reminder of the complex interplay between the visual system and other neurological or cognitive processes and systems: it is essential to look beyond purely ocular/visual factors in investigations of the aetiology of refractive error.

## Conclusion

The UKBB study has been able to deliver a high quality and highly powered cross-sectional dataset on refractive error by embedding an ophthalmic component within a generic epidemiological study. The findings of this study confirm refractive error as a significant public health issue for the ethnically diverse and ageing UK population. They serve to provide contemporary data that have been lacking for planning services and health economic modelling and as the baseline for monitoring secular trends. Further investigation of risk factors is necessary to inform strategies for prevention.

## Supporting Information

S1 Fig Flowchart of participation. (PDF)

S1 Table Comparison of subjects included in or excluded from analyses. (PDF)
