# Supplementary figures and images for: Frequency and Distribution of Refractive Error in Adult Life: Methodology and Findings of the UK Biobank Study
Source: PLoS One. 2015 Oct 2;10(10):e0139780. doi: 10.1371/journal.pone.0139780 (PMC4591976; doi:10.1371/journal.pone.0139780)

**S1 Fig. Flowchart of participation**

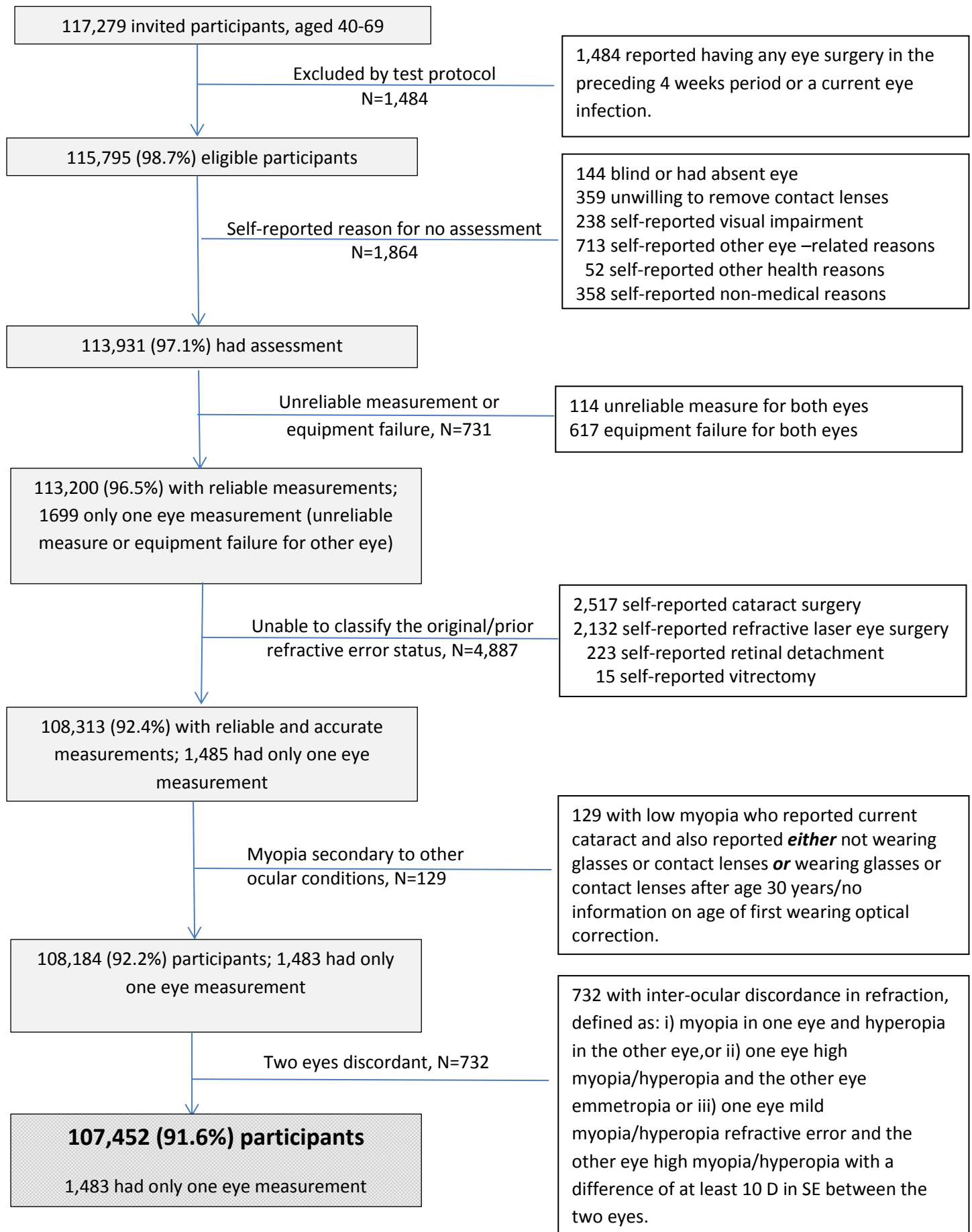

Supplement: S1 Fig — (PDF) [file pone.0139780.s001.pdf]
