# Supplementary material for: Frequency and Distribution of Refractive Error in Adult Life: Methodology and Findings of the UK Biobank Study
Source: PLoS One. 2015 Oct 2;10(10):e0139780. doi: 10.1371/journal.pone.0139780 (PMC4591976; doi:10.1371/journal.pone.0139780)
Supplement: S1 Table — (PDF) [file pone.0139780.s002.pdf]

S1 Table. Comparison of subjects included in or excluded from analyses

	Included in frequency analysis N=107,452	Excluded from frequency analysis ** N=9827	P value	Excluded from analysis of associations N=9095
	n (%)	n (%)		n (%)
Age band			<0.001	
40-49	24,782 (23.1%)	1802 (18.3%)		1668 (18.3%)
50-59	34,853 (32.4%)	2694 (27.4%)		2478 (27.3%)
60-69	47,817 (44.5%)	5331 (54.3%)		4949 (54.4%)
Gender			0.633	
Male	49,000 (45.6%)	4443 (45.2%)		4109 (45.2%)
Female	58,452 (54.4%)	5384 (54.8%)		4986 (54.8%)
Highest educational qualification *			<0.001	
No qualification	15,776 (14.9%)	1609 (16.7%)		1521 (17.2%)
O level	28,275 (26.7%)	2479 (25.8%)		2296 (25.8%)
A level	19,162 (18.1%)	1698 (17.7%)		1566 (17.6%)
Higher level	42,773 (40.4%)	3833 (39.9%)		3511 (39.4%)
Unknown	1466 (-)	208 (-)		201 (-)
Accommodation tenure			<0.001	
Rent from council	7301 (6.9%)	782 (8.2%)		732 (8.3%)
Rent from private	4351 (4.1%)	380 (4.0%)		354 (4.0%)
Own with mortgage	37,858 (35.9%)	3035 (31.7%)		2808 (31.7%)
Own	55,840 (53.0%)	5376 (56.2%)		4964 (56.0%)
Unknown	2102 (-)	254 (-)		237 (-)
Ethnicity			<0.001	
White	95,791 (89.8%)	8482 (87.4%)		7826 (87.2%)
Mixed	974 (0.9%)	90 (0.9%)		82 (0.9%)
Asian/Asian British	4032 (3.8%)	494 (5.1%)		466 (5.2%)
Black/Black British	3759 (3.5%)	400 (4.1%)		378 (4.2%)
Chinese	490 (0.5%)	47 (0.5%)		46 (0.5%)
Other	1627 (1.5%)	192 (2.0%)		181 (2.0%)
Unknown	779 (-)	122 (-)		116 (-)

* No qualifications, State school examinations at 16 years of age (‘O’ levels), at 18 years (‘A’ levels) or University/other professional qualification

**additional exclusion of those with discordant refractive errors in right/left eyes
